# Integrated care: implementation issues for ‘countries in transition’

**Published:** 2012-09-04

**Authors:** George Boulton

**Affiliations:** International Consultant and Lecturer on Health System Policy, Finance and Management, Former NHS CEO, Cardiff, Wales, UK

**Keywords:** countries in transition, integration, implementation, obstacles, minimisation coping strategies

## Abstract

**Purpose:**

Exploration of some major obstacles to integrated care in ‘countries in transition’ in central/eastern Europe.

**Context:**

‘Integration’ is virtually a universal policy aim of European ‘transition’ countries. Seduced by the prospect of increased value from scarce resources, governments strive to transform traditional compartmentalised ‘curative’ health systems, into more integrated systems focused on population health improvement and effective chronic disease management. Theory is commendable; practice far from straightforward.

**Data sources:**

Experience and data from implementing health system planning, financing and management reforms in 12 European ‘transition’ countries.

**Case description:**

Assessment of major obstacles to systemic change to integrated care delivery models.

**Conclusions:**

Key implementation issues can be discussed under the following headings:

- Legacy of traditional policy, professional and power systems
- Adherence to ‘prescriptive’ legislative styles
- Mismatch of health financing and manpower strategies
- Massive underinvestment in human resource development
- Rigid traditional organisational models and preferences
- Weak and fragmented planning and knowledge systems
- Powerful perverse countervailing influences
- Failure to grasp the complexities of major social system change
- Leadership and management deficits.

**Discussion:**

The ethos of ‘old style’ compartmentalised European health systems is deeply engrained. Systemic integrated care, needs coherent, complex, multi-faceted culture change—societal, professional and managerial—facing up to a number of major obstacles. These obstacles are analysed under the above eight headings, and some suggestions are advanced to minimise their impact on progress.

